# Optimized Model of Cerebral Ischemia *In situ* for the Long-Lasting Assessment of Hippocampal Cell Death

**DOI:** 10.3389/fnins.2017.00388

**Published:** 2017-07-06

**Authors:** Oksana Rybachuk, Olga Kopach, Volodymyr Krotov, Nana Voitenko, Tatyana Pivneva

**Affiliations:** Department of Sensory Signaling, Bogomoletz Institute of PhysiologyKyiv, Ukraine

**Keywords:** organotypic hippocampal slice cultures, oxygen-glucose deprivation, cell death, neuronal excitability, synaptic transmission

## Abstract

Among all the brain, the hippocampus is the most susceptible region to ischemic lesion, with the highest vulnerability of CA1 pyramidal neurons to ischemic damage. This damage may cause either prompt neuronal death (within hours) or with a delayed appearance (over days), providing a window for applying potential therapies to reduce or prevent ischemic impairments. However, the time course when ischemic damage turns to neuronal death strictly depends on experimental modeling of cerebral ischemia and, up to now, studies were predominantly focused on a short time-window—from hours to up to a few days post-lesion. Using different schemes of oxygen-glucose deprivation (OGD), the conditions taking place upon cerebral ischemia, we optimized a model of mimicking ischemic conditions in organotypical hippocampal slices for the long-lasting assessment of CA1 neuronal death (at least 3 weeks). By combining morphology and electrophysiology, we show that prolonged (30-min duration) OGD results in a massive neuronal death and overwhelmed astrogliosis within a week post-OGD whereas OGD of a shorter duration (10-min) triggered programmed CA1 neuronal death with a significant delay—within 2 weeks—accompanied with drastically impaired CA1 neuron functions. Our results provide a rationale toward optimized modeling of cerebral ischemia for reliable examination of potential treatments for brain neuroprotection, neuro-regeneration, or testing neuroprotective compounds *in situ*.

## Introduction

Cerebral ischemia is the disease that causes the highest mortality or severe disability in patients of various ages. Ischemic lesion can lead to functional deficit through various neurodegenerative disorders, such as encephalopathy (Martinez-Biarge et al., 2012), Alzheimer disease (Pluta et al., 2013), neuropathy of brain nerves (Tahir et al., 2016), and others, raised from either acute neuronal damage or delayed cell death. A wide range of impairments has been reported to underlie ischemic damage of the brain, including mitochondria dysfunction and energy failure, oxidative stress and inflammation (Noraberg et al., 2005; Kalogeris et al., 2016), ionic disbalance and excitotoxicity resulted from the increased level of extracellular glutamate and calcium overload (Kirino, 2000; Hardingham and Bading, 2010; Kopach et al., 2016).

Among all the brain, the hippocampus is the most susceptible region to ischemic damage, with a selective loss of CA1 pyramidal neurons after cerebral ischemia (Kirino et al., 1986; Johnston, 2001; Northington et al., 2011). The ischemia-induced death of CA1 neurons results in memory loss (Xu et al., 2016) and severe cognitive disorders (Morán et al., 2017) that may develop shortly or with a delayed appearance following insult. Different experimental models have been developed aimed to elucidate the mechanisms of this phenomenon and possible neuroprotection against ischemic damage, those vary by managing of simulating cerebral ischemia *in vivo, in situ*, or *in vitro* as well as by a duration of ischemic conditions. Amongst those, a model of mimicking ischemic conditions in organotypic hippocampal slice cultures is the one that yields long-lasting assessment of CA1 ischemic damage using oxygen-glucose deprivation (OGD), the conditions taking place under transient ischemic insult, to perfectly simulate ischemic excitotoxicity *in situ* (Tasca et al., 2015). A capability for the feasibly applied combinations of molecular and genetic approaches makes organotypic slice cultures perfectly suited for studying the precise mechanisms of post-ischemic tissue damage (Cho et al., 2004; Bonde et al., 2005; Chip et al., 2013).

Over decades after the ischemic death of CA1 neurons had been established, studies were predominantly focused on a short-term CA1 neuronal death, which takes 2–3 days to become morphologically observed (Pamenter et al., 2012; Chip et al., 2013; Le et al., 2015; Secondo et al., 2015). However, much less attention has been paid toward ischemic impairments occurring later—within the time-frame of 1–3 weeks following lesion. This gap is, at least partially, due to limited at present methodological studies of experimental modeling the long-lasting post-ischemic impairments. The optimized model of cerebral ischemia for such long-termed assessment would provide a useful tool for examination of potential neuroprotection and/or cell-therapy implementation.

In this study, we examined different schemes of OGD for the long-lasting assessment of ischemic CA1 neuronal damage vs. irreversible cell death using both morphological and electrophysiological justifications.

## Materials and methods

### Organotypic hippocampal slice culture

Organotypic hippocampal slice cultures were prepared from the 8 to 9-day-old pups of FVB mice. Animals were used in accordance with the protocols approved by the Animal Care and Use Committee at Bogomoletz Institute of Physiology (Kyiv, Ukraine) and the Law of Ukraine on protection of experimental animals (N3447-IV, 21.02.2006). Pups were decapitated, brains were quickly removed and the hippocampi were dissected and placed in a cold medium containing 50% Minimal Essential Medium (MEM), 25% Hanks' balanced salt solution (HBSS), 5 mM Tris, 2 mM NaHCO3, 12.5 mM HEPES, 15 mM glucose, 1% Penicillin and Streptomycin (pH 7.3). Transverse slices were cut (350 μm thick) and placed on 0.4-μm membrane inserts (Sigma, Millicell®CM, Germany). Slices were maintained in culturing medium containing 50% MEM, 25% HBSS, 25% horse serum (HS), 2.5 mM Tris, 2 mM NaHCO3, 12.5 mM HEPES, 15 mM glucose, 1% Penicillin and Streptomycin at 35°C in 95% O_2_ and 5% CO_2_ according to Stoppini et al. (1991). Medium was replaced on the day 2 after plating and then two to three times in a week. After 1–2 weeks, slice thickness reached 200–250 μm and surface became clear of damaged cells.

### Oxygen-glucose deprivation (OGD)

Different schemes of OGD were tested out in this study (10- to 30-min duration). The organotypic slice cultures (1 week post-plating) were placed into the designed chamber filled with a medium of composition similar to culturing medium but containing 15 mM sucrose (instead of 15 mM glucose) equilibrated with 95% N_2_ and 5% CO_2_ (pH 7.4) for the period of time as indicated in the text. After termination of OGD, slice cultures were returned into culturing medium and maintained until used. The age-matched organotypic slices subjected to similar procedures of the same duration but in the culturing medium equilibrated with 95% O_2_ and 5% CO_2_ (without ischemic treatment) were used as control.

### Propidium iodide (PI) staining

For the assessment of cell death in organotypic hippocampal slice cultures the propidium iodide (PI) staining was used as described (Hassen et al., 2004; Raval et al., 2006). PI (5 μM) was supplemented to organotypic slice cultures 1 h before OGD. Imaging was performed at different time points: at 2, 4, 6, 12, 24, 48, and 72 h post-OGD, using a microscope equipped with fluorescent filters (for rhodamine, XSP-139A-TP, China) and digital camera (Canon Power Short G-6). Images were taken from the focal plane at the depth at least 50 μm below the tissue surface. The number of PI-positive cells was counted within CA1 area arbitrary defined (400 μm^2^).

### Immunohistochemistry

Immunohistochemistry was performed as described in details previously (Rybachuk et al., 2014). Briefly, organotypic hippocampal slice cultures were fixed with 4% paraformalgehyde (PFA) for overnight, then washed in 0.1 M phosphate buffer (PB), with the next blocking in 0.1 M PB containing 0.3% Triton X-100 and 0.5% bovine serum albumin. Slices were incubated with primary antibodies overnight at 4°C. The following antibodies were used: monoclonal mouse anti-neuronal nuclei (NeuN, 1:1,000; Chemicon, UK), monoclonal rabbit anti-β-tubulin III (1:300; Sigma, USA), polyclonal chiken anti-glial fibrillary acidic protein (GFAP, 1:1,500; Dakocytomation, Denmark), rabbit polyclonal antibodies anti-Iba-1 (1:1,000, Wako, Japan) and polyclonal rabbit anti-caspase-3 (1:200, Sigma, USA). Secondary antibodies were anti-mouse Alexa Fluor-555 (1:1,000, Invitrogen, USA), anti-rabbit Alexa Fluor-488 and anti-chicken Alexa fluor-647 (1:1,000, Invitrogen, USA). Slices were mounted on glass slides with DakoCytomation Fluorescent Mounting Medium (DacoCytomation, Denmark). Confocal imaging was performed using a FV1000-BX61WI laser scanning microscope (Olympus, Tokyo, Japan) within a focal plane at the depth at least 50 μm below the tissue surface. The amount of fluorescent-labeled cells (about one CA1 pyramidal cell layer) was counted as described above.

### Electrophysiology

The whole-cell recordings were taken from CA1 neurons in organotypic slice cultures at different time-points of tissue maintenance (from 1 to 4 weeks). Current-clamp and voltage-clamp recordings were performed using MultiClamp 700B amplifier (Axon Instruments, Molecular Devices, CA, USA) and an Olympus BX50WI microscope (Olympus, Japan) equipped with a 60 ×, NA 09 water-immersion objective and infrared optics. An organotypic slice culture was placed in an artificial cerebrospinal fluid (ACSF) containing (in mM) 124 NaCl, 1.6 KCl, 24 NaHCO_3_, 1.2 KH_2_PO_4_, 2.5 CaCl_2_, 1.5 MgCl_2_, 2 ascorbic acid, 10 glucose (pH 7.4) equilibrated with 95% O_2_ and 5% CO_2_ at room temperature. Cells were patched with the electrodes of a resistance of 3–5 MΩ filled with an internal solution containing (in mM) 133 K-gluconate, 5 NaCl, 0.5 MgCl_2_, 10 HEPES-Na, 2 MgATP, 0.1 GTP-Na and 0.5 EGTA (pH 7.2, osmolarity 290 mOsM). Cells displaying a leak current >100 pA were discarded. CA1 neurons were studied for the passive electrophysiological properties, including the resting membrane potential (V_rest_), capacitance (C_m_) and input resistance (R_in_), and for the active firing discharge. Neuronal firing was elicited by series of depolarizing currents of 50-ms duration injected with increased stimulus intensity (an increment of 30–60 pA). We analyzed the frequency of action potentials (AP) and parameters of the first AP spike for its amplitude, overshoot and the spike width at half-maximal amplitude. For the analysis Clampfit 10.3 software (Molecular Devices) and Origin Pro (Origin Lab, USA) were used.

Voltage-clamp recordings of spontaneous excitatory postsynaptic currents (sEPSCs) in CA1 neurons were performed at −70 mV and analyzed using Mini Analysis Program (Synaptosoft, Decatur, GA) as described in details previously (Kopach et al., 2015). Excitatory currents were distinguished from baseline noise by setting the appropriate parameters for each individual cell and eliminating any false-positive events. Synaptic currents were analyzed for the frequency of their occurrence and the amplitude.

### Statistical analysis

All data are presented as mean ± s.e.m. Student's two-tailed *t*-test (paired or unpaired) was used to determine statistical differences between different experimental groups where appropriate. The data sets for the recorded sEPSCs were probed for normality using the Shapiro–Wilk test. The data sets not normally distributed were compared using a non-parametric Mann–Whitney *U*-test; the results were presented as medians with interquartile ranges (IQR) with *n* referring to the number of cells analyzed. The Kolmogorov-Smirnov two-sample test (KS-test) was used to compare the distributions of tested parameters between groups. A *p* < 0.05 was considered as statistically significant for either test.

### Chemicals

Horse serum was purchased from PAA Laboratories (Canada). HEPES, penicillin and streptomycin were purchased from Invitrogen (USA). All other chemicals were from Sigma Chemical Co. (Deisenhofer, Germany).

## Results

### Prolonged OGD results in a prompt CA1 neuronal death and overwhelmed astrogliosis

**Figure 1:**
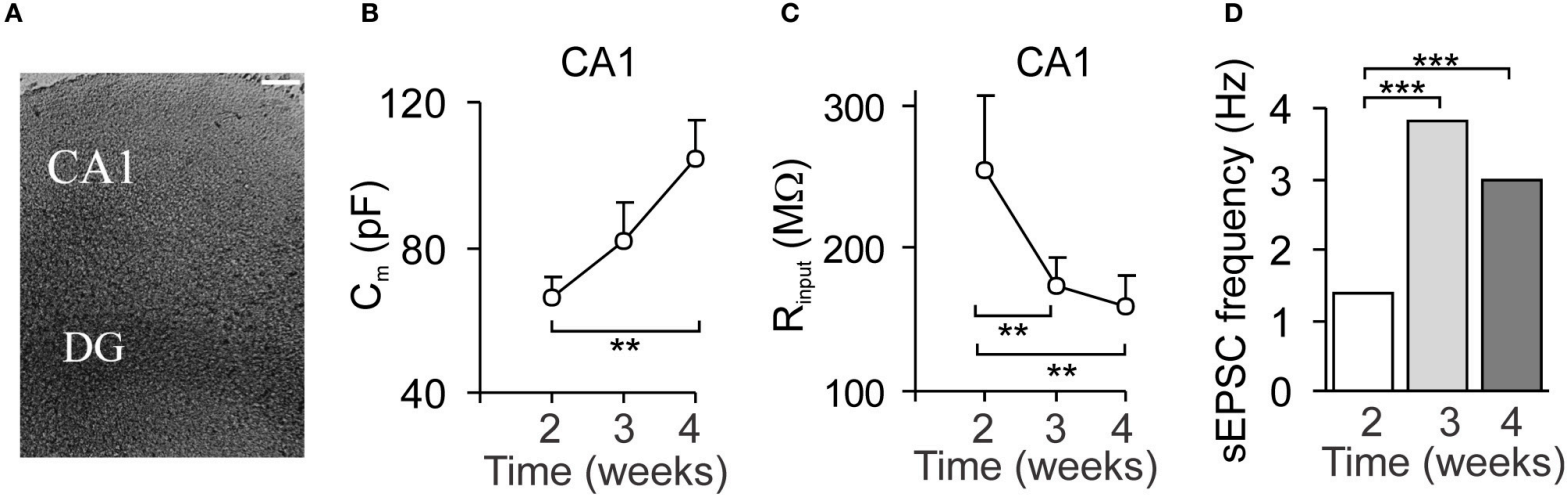
The organotypic hippocampal slice capability for long-term maintenance and neuronal maturation. **(A)** Phase-contrast image of an organotypic hippocampal slice 3 weeks after *in vitro* maintenance. Scale bar, 70 μm. **(B,C)** The time course of changes in the passive membrane properties of CA1 neurons: cell capacitance **(B)** and the input resistance **(C)**, over the time of tissue culturing. Data are mean with s.e.m. ^**^P < 0.01; ^***^p < 0.001 (unpaired *t*-test). **(D)** The time course of changes in synaptic activity of CA1 neurons evaluated as the frequency of spontaneous excitatory postsynaptic currents (sEPSC) over the time. Data are median values. ^***^P < 0.001 (Mann–Whitney *U*-test).

The organotypic hippocampal slice cultures displayed a capability for long-term maintenance (McBain et al., 1989; Stoppini et al., 1991; Van Bergen et al., 2003; Su et al., 2011). We enabled culturing of mouse hippocampal slices with the preserved characteristic morphology of the hippocampal tissue, including all cell layers, for at least 4 weeks of *in vitro* maintenance (Figure 1A). Electrophysiology has confirmed a functional viability of CA1 pyramidal neurons in organotypic slices, which maturated with the time of tissue culturing. We observed the progressively increased capacitance of CA1 neurons (66 ± 6 pF, *n* = 12 and 104 ± 11 pF, *n* = 9, *p* < 0.01 at weeks 2 and 4, respectively; Figure 1B); it was accompanied with the decreased membrane resistance (*p* < 0.01; Figure 1C), reflecting the increased membrane conductance. In line with this, a marker of cytoskeleton, β-tubulin III, demonstrated higher immunoreactivity in CA1 area with the time of slice culturing (data not shown). The voltage-clamp recordings of synaptic activity of CA1 neurons revealed the increased sEPSC frequency from the week 1–4 of slice culturing (Figure 1D).

**Figure 2:**
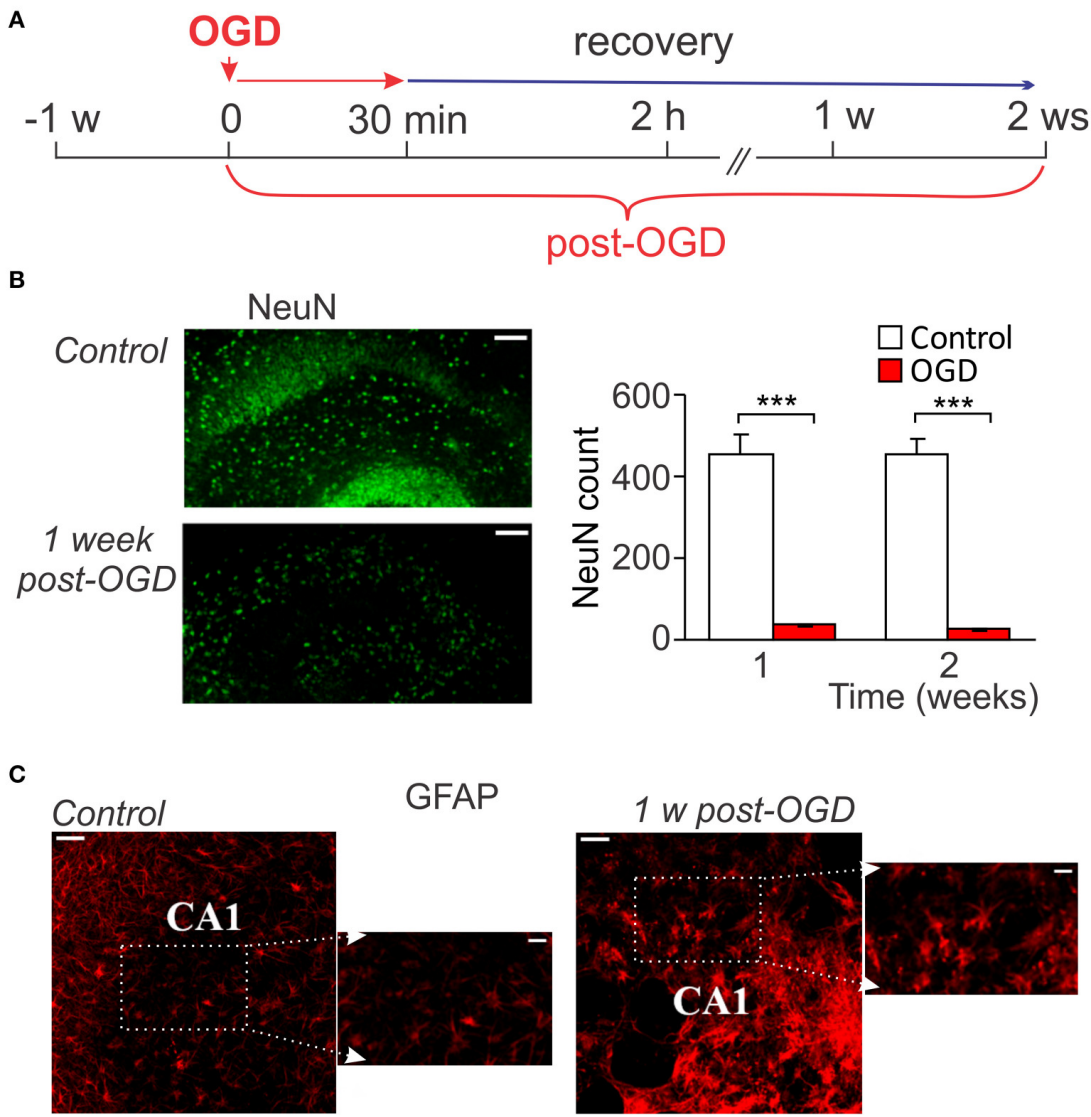
Neuronal death and astrogliosis following prolonged OGD. **(A)** Cartoon of an experimental modeling of prolonged ischemic conditions (30-min duration) in organotypic hippocampal slice cultures for long-term assessment of ischemic CA1 neuronal death. **(B)** Immunohistology with the neuronal marker, NeuN (left images), and its quantitative analysis in CA1 area in control (*n* = 7 slices) and after prolonged OGD (*n* = 3 slices exposed to 30-min OGD; right graphs). Scale bar, 100 μm. Data are mean with s.e.m. ^***^P < 0.001 (*t*-test). **(C)** Immunohistology with astrocytic marker GFAP in control organotypic slice and one exposed to prolonged OGD 1 week-post. Note a prominent outgrowth of GFAP-positive cells after OGD. Scale bar, 50 μm; insert, 20 μm.

A massive death of CA1 neurons following OGD of a prolonged duration (from 30 min to up an hour) has been evidenced within hours after re-oxygenation that lasted for the next few days (Lipton, 1999; Kirino, 2000; Rytter et al., 2003). However, the ischemic impairments during the later period of time (over weeks after induction of lesion) have not been explored. Therefore, we aimed to examine changes at the delayed time-frames—within 1–2 weeks after prolonged OGD (30-min duration; Figure 2A). Prolonged OGD resulted in severe tissue damage observed at this time-frame. Quantitative analysis of immunostaining with the neuronal marker, NeuN, revealed a dramatic drop in the number of NeuN-positive cells (viable neurons) in CA1 area: from ~457 (*n* = 7/slices) in control to ~37 (*n* = 3/slices, *p* < 0.001) at the week 1 post-OGD and from ~454 (*n* = 7) to ~25 (*n* = 3, *p* < 0.001) at the week 2, respectively (Figure 2B). Immunostaining with GFAP, a marker of astrocytes, demonstrated reactive astrogliosis, with astrocytic processes densely sprouted through CA1 area 1 week after prolonged OGD (Figure 2C). A massive neuronal death produced by OGD of 30-min duration resulted in severe histopathology: a loss of the tissue density and numerous cavities appeared within CA1 area (Figure 2C).

### Delayed CA1 neuronal death by a mild OGD

**Figure 3:**
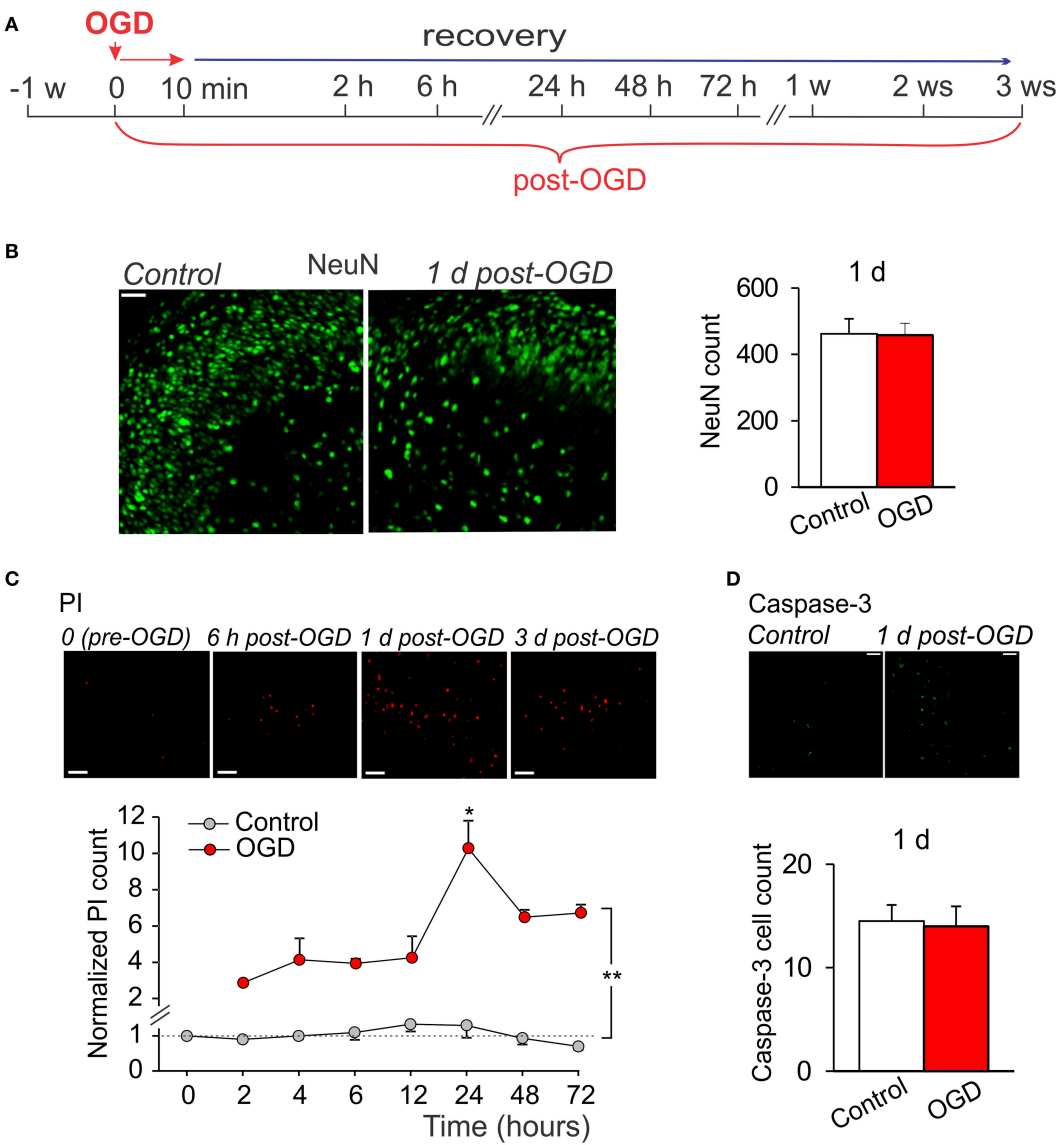
Mild OGD produces little acute effects on CA1 morphology. **(A)** Cartoon of an experimental modeling of mild OGD (10-min duration) in organotypic hippocampal slice cultures, showing time-scale for the assessments of acute, and delayed CA1 neuronal damage. **(B)** Immunohistology with the neuronal marker, NeuN (left images), and quantitative analysis of the NeuN-positive cells in CA1 area (right graphs) in control (*n* = 5 slices) and 1 d after induction of mild OGD (*n* = 13 slices exposed to 10-min OGD) demonstrate no acute changes between experimental groups. Data are mean with s.e.m. **(C)** Images of propidium iodide (PI) uptake in CA1 area in a slices exposed to mild OGD (upper raw) and the time course of changes in average number of PI-positive cells in control (*n* = 4 slices) and after mild OGD (*n* = 4 slices exposed to 10-min OGD; bottom). The number of PI-positive cells is normalized to control value (“0” time-point, before induction of OGD). ^**^P < 0.01 (unpaired *t*-test compared with the corresponding control). **(D)** Immunohistology with caspase-3 in CA1 area (top raw) and average number of the caspase-3-positive cells in control (*n* = 4 slices) and 1 d after mild OGD (*n* = 11 slices exposed to 10-min OGD) demonstrate no difference between groups (bottom). Scale bar, 50 μm for all panels. Data are mean with s.e.m.

Having observed a massive tissue degeneration within a week following prolonged OGD, we next modified experimental scheme by shortening a duration of slice exposure to OGD to 10 min (mild OGD; Figure 3A). For the assessment of time course of CA1 neuronal damage vs. a delayed cell death immunohistology with various morphological markers were performed at different time-points post-OGD: within several hours (acute changes) and then up to a few weeks (delayed impairments).

There were no changes in the amount of NeuN-positive cells in CA1 area in organotypic hippocampal slices exposed to a mild OGD (10-min duration) at 24 h, as compared to control (~462, *n* = 5/slices in control and ~457, *n* = 13/slices post-OGD; Figure 3B). However, the number of PI-positive cells increased in CA1 area (Figure 3C). The increase became significant at 2 h post-OGD (~two-fold, *p* < 0.01) and reached a peak level at 24 h (*p* < 0.01) but returned backwards on the day 2 and 3, at a level still higher than that in control (*n* = 4/slices, *p* < 0.01; Figure 3C). To validate whether this PI rise (indicating a drop in cell viability due to damaged membranes) is due to a programmed cell death initiated shortly by a mild OGD, we used immunostaining with caspase-3, the pro-apoptotic mediator which activation relates to a loss of hippocampal cell viability (Porter and Jänicke, 1999; Rami, 2003; Qi et al., 2004; Liu et al., 2013). However, no significant difference in the caspase-3 immunoreactivity was found between control (*n* = 4/slices) and 24 h post-OGD (*n* = 11/slices, *p* > 0.8; Figure 3D), suggesting that mild OGD does not cause acute CA1 neuronal death.

**Figure 4:**
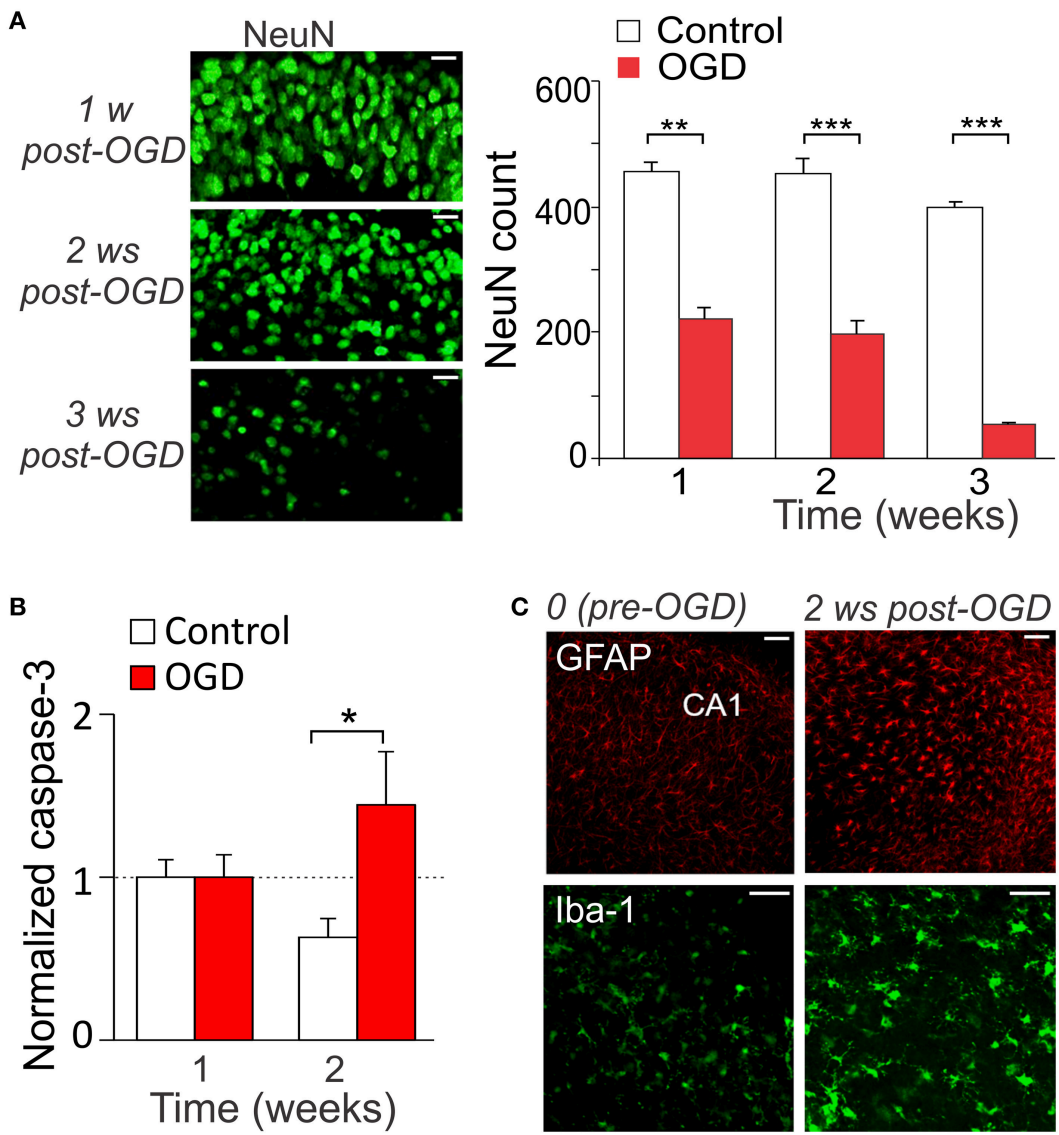
Mild OGD triggers the delayed CA1 neuronal death. **(A)** Immunohistology with the neuronal marker, NeuN (left images), and quantitative analysis of the NeuN-positive cells in CA1 area in control (*n* = 7 slices) and after mild OGD (*n* = 7 slices exposed to 10-min OGD; right graphs). Scale bar, 20 μm. Data are mean with s.e.m. **(B)** Quantitative analysis of the caspase-3-positive cells in control (*n* = 4) and 1–2 weeks post-OGD (*n* = 10 slices exposed to 10-min OGD). The numbers of fluorescent-labeled cells are normalized to the correspondent value at 1 week post-OGD. **(C)** Immunohistology with the astrocytic marker, GFAP, and the microglial marker, Iba-1, before and 2 weeks after mild OGD. Scale bar, 50 μm. ^*^P < 0.05, ^**^p < 0.01, ^***^p < 0.001 (unpaired *t*-test compared with corresponding control as indicated).

We have observed the delayed CA1 neuronal death, which appeared later on (over weeks) after induction of mild OGD. There was a dramatic drop in the NeuN-positive cells in CA1 area at the time-frame 1–3 weeks post-OGD (by ~51%, *n* = 7, *p* < 0.01 at the week 1 and by ~57% and ~87%, *p* < 0.001 at the weeks 2 and 3, respectively; Figure 4A). Consistent with this, the caspase-3 immunoreactivity dramatically increased from the week 2 post-OGD (by ~121%, *n* = 10, *p* < 0.01; Figure 4B). Promoted gliosis was also observed at this time-frame, revealed by the increased immunoreactivity for GFAP (marker of astrocytes) and Iba-1 (marker of microglial cells; Figure 4C). The main features of reactive astrogliosis were hypertrophy of astrocytic processes and soma (Cho et al., 2013) whereas microgliosis revealed cells of ameboid form, with increased cell body and fewer ramified processes (Rappert et al., 2004).

### Impairments in CA1 neuronal function by a mild OGD

**Figure 5:**
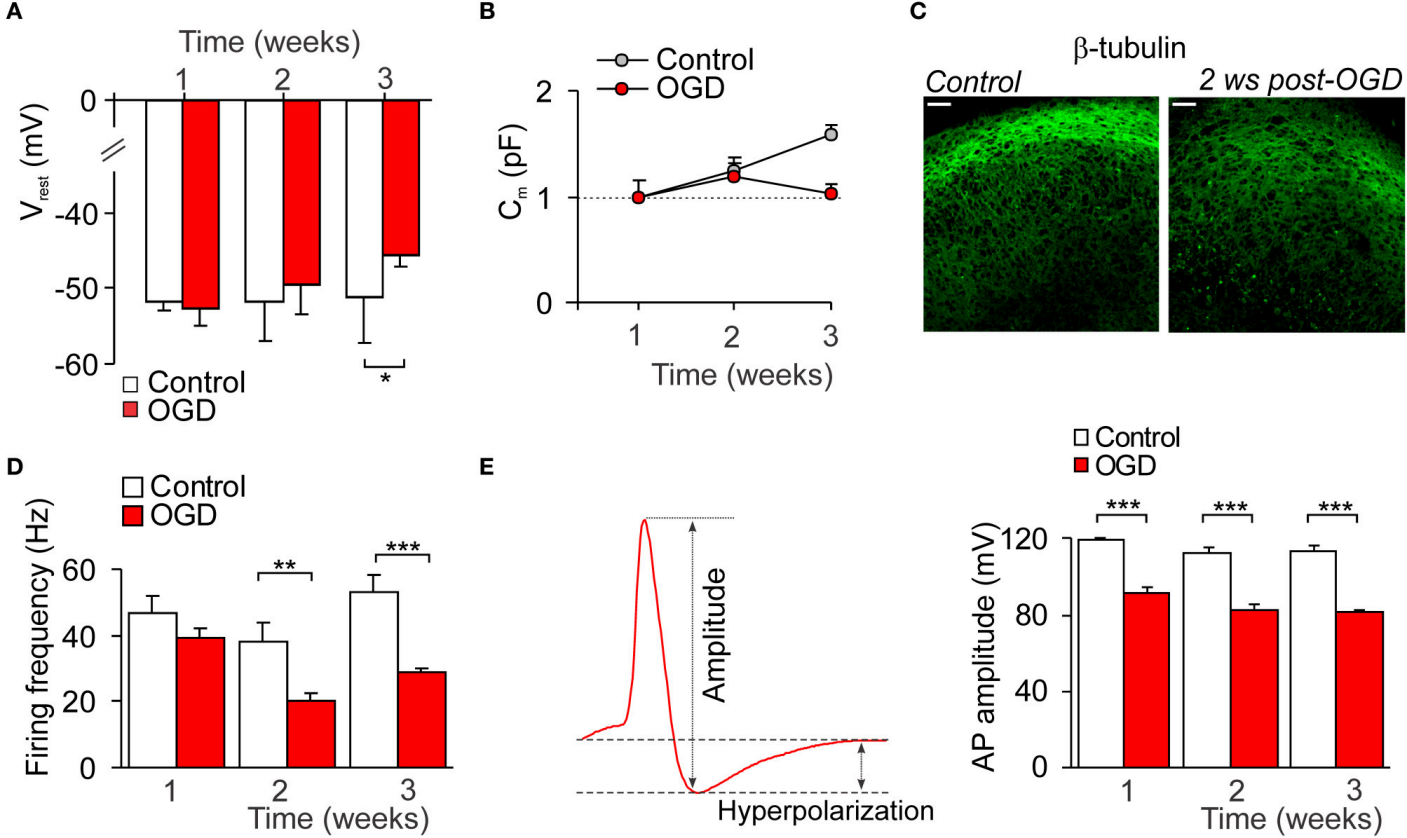
Impairments in the CA1 neuronal excitability following mild OGD. **(A,B)** The time course of changes in the resting membrane potential **(A)** and capacitance **(B)** of individual CA1 neurons following mild OGD. **(C)** Immunohistology demonstrates the reduced immunoreactivity for β-tubulin III in CA1 area 2 weeks after mild OGD. Scale bar, 50 μm. **(D)** The time course of changes in the firing frequency of CA1 neurons following mild OGD. **(E)** Analysis of the first action potential spike (left image) revealed a drop in the spike amplitude in CA1 neurons by a mild OGD (right histogram). All data are mean with s.e.m. ^*^P < 0.05, ^**^p < 0.01, ^***^p < 0.001 (unpaired *t*-test compared with corresponding control, as indicated).

Electrophysiology has been performed to figure out the delayed impairments of CA1 neuron function following mild OGD. Whole-cell recordings of CA1 neurons revealed a progressive neuronal depolarization over the delayed period of time after induction of mild OGD (a drop in the resting membrane potential by ~5%, *n* = 12 on the week 2 and by ~11%, *n* = 7 on the week 3, *p* < 0.05; Figure 5A). Also, there was the OGD-induced decline in the capacitance of CA1 neurons increased with a time of slice culturing (neuronal maturation; Figure 5B). In line with this, β-tubulin III immunoreactivity in CA1 area became faint post-OGD (Figure 5C).

The current-clamp recordings demonstrated the reduced firing discharge of CA1 neurons following mild OGD. A decrease in the firing frequency was observed from the week 1 post-OGD (by ~17%, *n* = 10, *p* = 0.087), being ever larger later on (by ~48%, *n* = 13, *p* < 0.01 at the week 2 and by ~46%, *n* = 8, *p* < 0.001 at the week 3; Figure 5D). There were changes in the parameters of a single AP spike (Figure 5E left). The spike amplitude was reduced by ~24% (from 119.5 ± 0.8 pA, *n* = 10 in control to 91.1 ± 5.7 pA, *n* = 10 at 1 week post-OGD, *p* < 0.001; Figure 5E) and the after-hyperpolarization amplitude dropped by ~43% (*p* < 0.01; data not shown).

**Figure 6:**
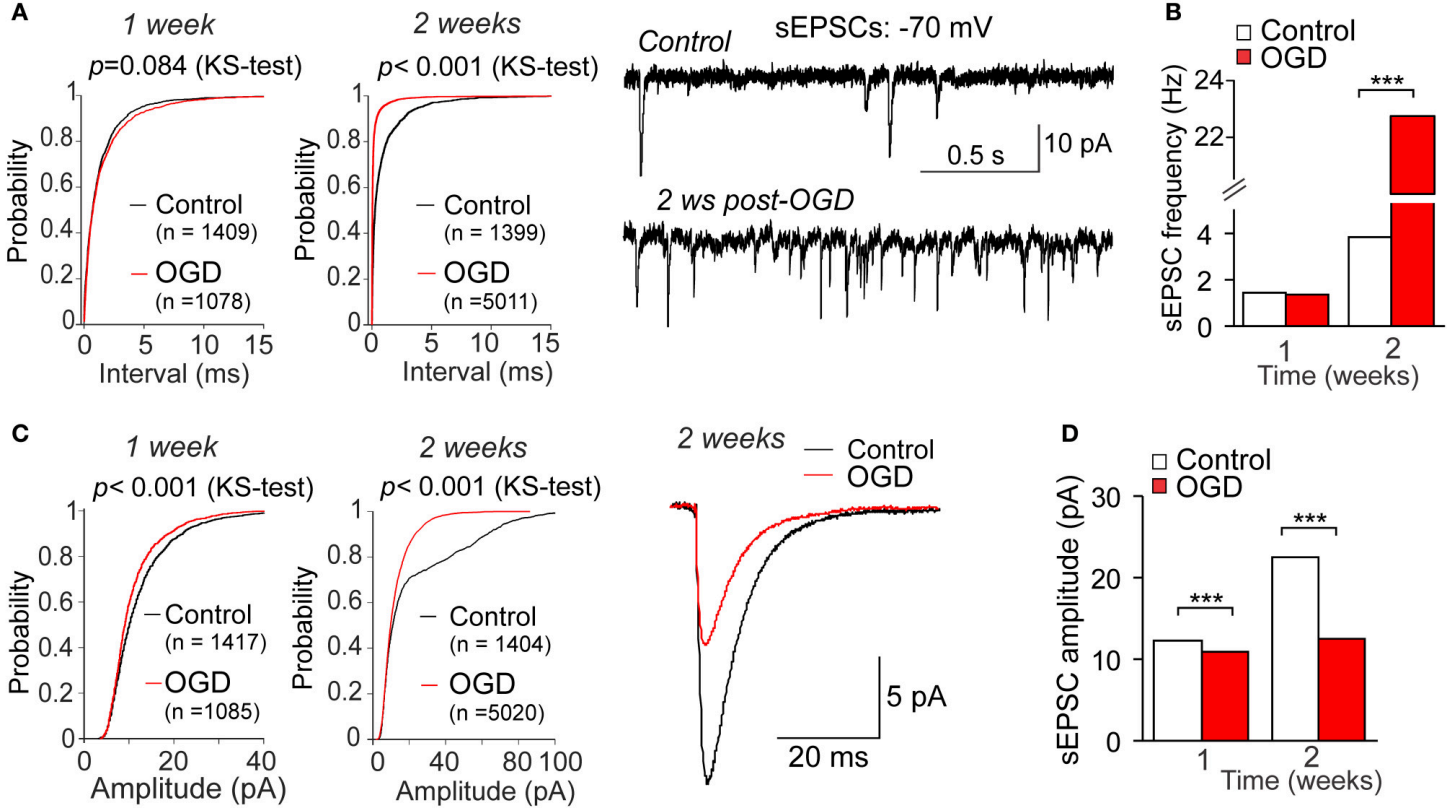
Changes in synaptic transmission in CA neurons by a mild OGD. **(A)** Voltage-clamp recordings in CA1 neurons held at −70 mV (right) demonstrate the OGD-induced changes in the frequency of spontaneous excitatory postsynaptic currents (sEPSCs) within the delayed time-frame after induction of mild OGD. Left, cumulative probability plots for the inter-event interval of sEPSCs in control and the weeks 1 and 2 post-OGD. The number of events analyzed for experimental groups and the Kolmogorov–Smirnov (KS)-test significance are indicated. **(B)** Summary for the median frequency of sEPSCs in control and post-OGD. ^***^P < 0.001 (the KS- and the Mann–Whitney *U*-tests). **(C)** Averaged sEPSCs in CA1 neurons in control and 2 weeks post-OGD (right) and the cumulative probability plots for the sEPSC amplitude at the weeks 1 and 2 after induction of mild OGD (left) demonstrate the gradually reduced current amplitude by a mild OGD. The number of events analyzed for experimental groups and the KS-test significance are indicated. **(D)** Summary for the median current amplitude in control and post-OGD. ^***^P < 0.001 (the KS-test).

The voltage-clamp recordings of sEPSCs demonstrated the delayed changes in synaptic transmission in CA1 neurons following mild OGD (Figure 6A). The frequency of sEPSCs dramatically increased within weeks post-OGD (from 3.82 Hz in control to 22.73 Hz at the week 2 after mild OGD, increase in ~six-fold; Figures 6A,B). The median inter-event interval of sEPSCs was 262 ms in control, but 44 ms post-OGD (*p* < 0.001, the KS- and *U*-tests). In the meantime, the amplitude of postsynaptic currents was markedly reduced (by ~44%, a drop from 22.5 pA in control to 12.5 pA after OGD; *p* < 0.001, the KS-test; Figures 6C,D).

## Discussion

This study aimed to optimize experimental modeling of cerebral ischemia in hippocampal organotypic slice cultures for the long-lasting assessment of ischemic damage/death of CA1 neurons. Mouse hippocampal organotypic slice cultures were maintained for at least 4 weeks *in situ* with the preserved tissue morphology and functional neurophysiology, including maturing of CA1 neurons. We managed long-termed assessment (within weeks) of ischemic impairments in CA1 neurons to establish the time course of neuronal damage vs. a selective CA1 neuronal death under different schemes of OGD for experimental modeling of cerebral ischemia in organotypic slice cultures. Prolonged OGD (30-min duration) produced a massive cell death, reactive astrogliosis and severe tissue degeneration within a week after induction of ischemic conditions. In a similar tissue preparation, mild OGD (10-min duration) triggered a programmed (apoptotic) CA1 neuronal death with a delayed appearance (within 2 weeks) that was associated with impairments in CA1 neuron function observed from week 1 post-OGD.

Amongst, various experimental models for mimicking cerebral ischemia *in vivo, in vitro*, or *in situ* to elucidate the mechanisms of ischemic damage and possible neuroprotection, the organotypic slice cultures have been often a subject of preference (Tasca et al., 2015). This is due to a wide range of advantages. First, unlike the dissociated cell cultures, the tissue cultures remain the preserved morphology with all cell types and layers along to retained native local connectivity and synaptic organization. Second, maintaining of tissue *in vitro* for a long term does not require high cost of animal housing and treatments *in vivo*. Third, a desired combination of genetic and cell-targeted interventions can be feasibly applied into brain tissue, enabling studies of the fine molecular mechanisms of brain damage, neuroprotection, and neurorepair. Here we validate that the mouse hippocampal organotypic slice cultures are capable for the long term maintenance (at least, 4–5 weeks). This was demonstrated by morphology (either NeuN or PI markers for cell viability remained at a steady level over the tested period) and the whole-cell recordings, which revealed furthermore a progressive maturation of CA1 pyramidal neurons due to the increased capacitance together with the input resistance (a hallmark of the membrane ion channel conductance), and promoted synaptic activity of CA1 neurons with a time of tissue culturing. Consistently, a capability of hippocampal organotypic slice cultures had been demonstrated for either mouse or rat preparations by others (Wise-Faberowski et al., 2009; Su et al., 2011; Kim et al., 2013). This provides a background for optimizing experimental modeling of cerebral ischemia in order to extend the onset of a prompt neuronal death and irreversible tissue degradation shortly after lesion, enabling long-termed testing of ischemic neurodegeneration and neuroprotection.

A selective death of CA1 pyramidal neurons, which has been established to last by 24–48 h post-OGD, was discovered over decades ago and firmly evidenced by different approaches (Lipton, 1999; Kirino, 2000; Sullivan et al., 2002). After years of investigating neuronal vulnerability to ischemic damage through different models of mimicking ischemic insults *in situ*, it has been emerged that the degree of neuronal damage and ultimate cell death strictly depend on severity of such modeling (e.g., a duration of lesion). Our results of a sharp neuronal loss and overwhelmed astrogliosis within destructive CA1 area by prolonged OGD (30 min duration) also argue for that, being fully consistent with previous reports of a massive cell death by OGD lasting up to an hour (Graulich et al., 2002; Rytter et al., 2003; Jung et al., 2004; Le et al., 2015) and astrogliosis (Cho et al., 2013; Honsa et al., 2014). In contrast to 30-min OGD, mild OGD (10-min duration) produced a little destruction to the hippocampal tissue, justified by a steady level of both NeuN-positive and PI-positive cells shortly after induction of mild OGD, similar to other studies (Matsuzaki et al., 1997; Lecoeur, 2002). Nevertheless, a rise in the number of PI-positive cells at 24 h post-OGD indicates acute drop in a cell viability (Rytter et al., 2003; Montero et al., 2007), which has been declined by the next day. Despite the PI uptake became a routinely used marker of cell death by taking place only in damaged cells, it is rather a hallmark of necrotic cell death (MacKlis and Madison, 1990; Noraberg et al., 1999) whereas other markers, specific to programmed (apoptotic) cell death mechanisms, should be considered (Kanduc et al., 2002). Therefore, we used immunostaining with caspase-3 catalyzing a cleavage of impaired proteins/organelles through activation of extrinsic or intrinsic (mitochondrial) pathways (Porter and Jänicke, 1999; Troy and Salvesen, 2002; Ghavami et al., 2009) to relate a loss of cell viability to a programmed cell death initiated by a mild OGD. We found no changes in the caspace-3 immunoreactivity in CA1 area on the day 1 that is consistent with unaltered number of the NeuN-positive cells at this time-point. However, the caspase-3 immunoreactivity increased after 2 weeks post-OGD that is consistent with a dramatic drop in the NeuN-positive cells at this time-frame, indicating the delayed CA1 neuronal death.

Boosted gliosis has been observed at the delayed time-frames after mild OGD. Both astrogliosis and microglia activation were detected with specific markers in organotypic slice cultures exposed to mild OGD. Astrocytes and microglia are known for their prompt activation after induction of cerebral ischemia with a particular role in initiating the development of pathogenesis by releasing cytokines and chemokines (Li et al., 2008; Sofroniew and Vinters, 2010; Ke et al., 2014; Matsui et al., 2014; Barakat and Redzic, 2015; Lv et al., 2016). Astrocytes produce pro-inflammatory factors (chemokine ligands, interleukin (IL)-1b, IL-6, etc. (Colombo and Farina, 2016), but may also serve with anti-inflammatory properties (Liu and Chopp, 2016; Sofroniew, 2017). Microglial cells also produce inflammatory mediators, including interleukins, tumor necrosis factor (TNF), and matrix metalloproteinases (MacRez et al., 2011; Taylor and Sansing, 2013), they are principal immunocompetent cells of the brain with a phagocytic activity (Bechmann and Nitsch, 2000; Pan et al., 2017) but can also play a neuroprotective role by producing anti-inflammatory factors (Vinet et al., 2012; Hu et al., 2016).

Functional impairments of individual CA1 neurons after mild OGD accompanied morphological changes. Both passive membrane properties and neuronal firing of CA1 neurons were changed at the delayed time-frame post-OGD, with the changes in excitatory transmission. Whole-cell recordings revealed a drop in the resting membrane potential of CA1 neurons and a decline of neuronal maturation (increased capacitance) after mild OGD. The hypoxia/ischemia-induced membrane dysfunction of CA1 pyramidal neurons has been established long time ago (Hansen and Olsen, 1980; Isagai et al., 1999), showing neuronal depolarization as an immediate response to slice exposure into ischemic medium *in vitro*, with the membrane potential reaching 0 mV within minutes. Numerous studies demonstrated using sharp electrodes or intracellular recordings that acute response of hippocampal pyramidal neurons to OGD consists of a few phases, which vary depending on a duration of slice exposure to OGD (Fujiwara et al., 1987; Cherubini et al., 1989; Dzhala et al., 2001). After period of ischemic depolarization, a period of post-ischemic hyperpolarization is typically seen with a depression of neuronal activity, followed by the next depolarization and neuronal dysfunctions. Our data demonstrated no significant changes in the resting membrane potential of CA1 neurons at the week 1 after mild OGD, but neuronal depolarization at the week 2, which gradually developed later on. Ischemic depolarization is Ca^2+^-dependent, mediated by a massive Ca^2+^ influx via activated NMDA receptors, causing irreversible neuronal damage and increased excitatory transmission (Zhang et al., 2008; Hardingham and Bading, 2010; Zanelli et al., 2015). In fully agreement with this, depolarization of CA1 neurons was associated with the increased excitatory transmission at the week 2 after mild OGD. The frequency of sEPSCs was dramatically increased (i.e., ~six times), reflecting an increase in presynaptic glutamate release that is due to the excessive glutamate release and activation of postsynaptic glutamate receptors in the conditions of extensive metabolic stress and excitotoxicity produced by OGD (Liu and Zukin, 2007; Hardingham and Bading, 2010). Notably, the EPSC amplitude was decreased post-OGD, indicating that postsynaptic changes also contribute to pathophysiological transmission at the delayed time-frames by mild OGD. This is consistent with changes in postsynaptic NMDA receptors (Stanika et al., 2010; Chung et al., 2016) and AMPARs (Noh et al., 2005; Liu and Zukin, 2007), resulted from altered subunit composition in CA1 neurons, that promoted selective death of this neuronal population.

Summarizing, our results argue that mild OGD (10-min duration) represents a reliable approach for experimental modeling of cerebral ischemia *in situ*, providing a window for long-lasting assessment of neuronal impairments in hippocampal tissue. This experimental approach is useful for further investigations of the precise mechanisms of delayed ischemic impairments and continuing treatments for potential brain neuroprotection and neuro-regeneration.

## Ethics statement

This study was carry out in accordance with the protocols approved by the Animal Care and Use Committee at Bogomoletz Institute of Physiology (Kyiv, Ukraine) and the Law of Ukraine on protection of experimental animals (N3447-IV, 21.02.2006).

## Author contributions

OR: Slice preparation, design of the oxygen-glucose deprivation, immunohistochemical experiments, data analysis, and interpretation. OK: Design and electrophysiological recordings, data analyses and interpretation, manuscript preparation. VK: The sEPSC detection and statistical analysis. NV: Conceiving the study, manuscript revision. TP: Research concept and conceiving the study, data interpretation, manuscript preparation. All authors read and approved the final manuscript.

### Conflict of interest statement

The authors declare that the research was conducted in the absence of any commercial or financial relationships that could be construed as a potential conflict of interest.
